# Alirocumab, evinacumab, and atorvastatin triple therapy regresses plaque lesions and improves lesion composition in mice[S]

**DOI:** 10.1194/jlr.RA119000419

**Published:** 2019-12-16

**Authors:** Marianne G. Pouwer, Elsbet J. Pieterman, Nicole Worms, Nanda Keijzer, J. Wouter Jukema, Jesper Gromada, Viktoria Gusarova, Hans M. G. Princen

**Affiliations:** Metabolic Health Research,* The Netherlands Organization of Applied Scientific Research (TNO), Gaubius Laboratory, Leiden, The Netherlands; Department of Cardiology† Leiden University Medical Center, Leiden, The Netherlands; Einthoven Laboratory for Experimental Vascular Medicine,§ Leiden University Medical Center, Leiden, The Netherlands; Regeneron Pharmaceuticals,** Tarrytown, NY

**Keywords:** atherosclerosis, drug therapy/hypolipidemic drugs, macrophages/monocytes, apolipoproteins, antibodies, regression, APOE*3-Leiden.CETP mice, angiopoietin-like proteins, PCSK9;, cardiovascular disease

## Abstract

Atherosclerosis-related CVD causes nearly 20 million deaths annually. Most patients are treated after plaques develop, so therapies must regress existing lesions. Current therapies reduce plaque volume, but targeting all apoB-containing lipoproteins with intensive combinations that include alirocumab or evinacumab, monoclonal antibodies against cholesterol-regulating proprotein convertase subtilisin/kexin type 9 and angiopoietin-like protein 3, may provide more benefit. We investigated the effect of such lipid-lowering interventions on atherosclerosis in APOE*3-Leiden.CETP mice, a well-established model for hyperlipidemia. Mice were fed a Western-type diet for 13 weeks and thereafter matched into a baseline group (euthanized at 13 weeks) and five groups that received diet alone (control) or with treatment [atorvastatin; atorvastatin and alirocumab; atorvastatin and evinacumab; or atorvastatin, alirocumab, and evinacumab (triple therapy)] for 25 weeks. We measured effects on cholesterol levels, plaque composition and morphology, monocyte adherence, and macrophage proliferation. All interventions reduced plasma total cholesterol (37% with atorvastatin to 80% with triple treatment; all *P* < 0.001). Triple treatment decreased non-HDL-C to 1.0 mmol/l (91% difference from control; *P* < 0.001). Atorvastatin reduced atherosclerosis progression by 28% versus control (*P* < 0.001); double treatment completely blocked progression and diminished lesion severity. Triple treatment regressed lesion size versus baseline in the thoracic aorta by 50% and the aortic root by 36% (both *P* < 0.05 vs. baseline), decreased macrophage accumulation through reduced proliferation, and abated lesion severity. Thus, high-intensive cholesterol-lowering triple treatment targeting all apoB-containing lipoproteins regresses atherosclerotic lesion area and improves lesion composition in mice, making it a promising potential approach for treating atherosclerosis.

Atherosclerosis is the main cause of CVD, and the annual number of deaths from CVD is predicted to rise from 17.5 million in 2012 to 22.2 million by 2030 (1). In addition to lifestyle changes (2), lipid lowering has proven to be highly effective in reducing CVD, as every 1 mmol/l reduction in LDL-C is associated with a 23% CVD risk reduction (3). Because most patients at CVD risk are treated after development of atherosclerosis, therapies that regress pre-existent lesions are warranted.

Currently, statins are the “golden standard” to lower LDL-C and to reduce CVD risk, but monotherapy with statins remains suboptimal as the achieved regression is modest, reflected by the small reductions in plaque volume (0.3–1.2% per year) (4, 5). Furthermore, plaque regression is only seen in those patients with LDL-C reductions of >40% (6, 7), or at plasma LDL-C levels below 78 mg/dl (2.0 mmol/l) (5, 7), while a subgroup of patients still does not reach their LDL-C goals. Notably, the magnitude of regression is correlated with the percentage of LDL-C reduction (5, 6), indicating the potential for further lipid lowering. In this context, dual lipid-lowering therapies using ezetimibe or inhibition of proprotein convertase subtilisin/kexin type 9 (PCSK9) on top of a statin further reduce plaque volume relative to monotherapy with statins (5). While currently available therapies aim mostly to decrease plasma LDL-C, remnant cholesterol and triglyceride (TG) levels are considered to be an important residual risk factor for CVD as well (8, 9), and non-HDL-C appears to be superior to LDL-C in CVD risk estimation (10). Essentially, the clinical benefit of lowering TGs and LDL-C may be proportional to the absolute change in apoB, implicating that all apoB-containing lipoproteins have approximately the same effect on the risk of CVD per particle (11). Therefore, novel high-intensive lipid-lowering or combination therapies targeting all apoB-containing lipoproteins may provide additional benefit to regress atherosclerosis and further reduce clinical events.

Because the severity and progression of coronary atherosclerosis are associated with adverse cardiovascular outcomes (4, 12), the modest reduction in plaque volume achieved by statins cannot fully explain the reduced CVD risk, suggesting an important role for improved lesion stability (5, 13, 14). Animal models represent an opportunity to study plaque composition during regression. However, many mouse models have limited translational capability due to lack of responsiveness to lipid-lowering treatment (14). In this study, we utilized the APOE*3-Leiden.CETP mouse, a well-established model for hyperlipidemia, characterized by increased plasma cholesterol and TG levels, that resembles lipoprotein metabolism of patients with familial dysbeta­lipoproteinemia (FD) (15). The APOE*3-Leiden.CETP mice are prone to atherosclerosis development (15) and respond well to hypolipidemic drugs (16–21). FD or type III hyperlipoproteinemia presents with elevated levels of plasma cholesterol and TGs and an increased ratio of cholesterol to TGs in the VLDL and IDL fractions, resulting in the appearance of β-VLDL particles and development of premature atherosclerosis (22). While normal wild-type mice have a very rapid clearance of apoB-containing lipoproteins, APOE*3-Leiden.CETP mice have impaired clearance and an increased TG level and, thereby, mimic the slow clearance of these particles observed in humans, particularly in patients with FD. Importantly, as compared with the widely used hyperlipidemic and atherogenic apoE- and LDLR-deficient (*Apoe*^−/−^ and *Ldlr*^−/−^) mice, the APOE*3-Leiden.CETP mice possess an intact but delayed apoE-LDLR-mediated clearance, which is required for the proper response to hypolipidemic drugs (15–21, 23–27).

Alirocumab is a fully human monoclonal antibody to PCSK9 that reduces the risk of recurrent ischemic cardiovascular events in patients with acute coronary syndrome when administered on top of atorvastatin (28). Evinacumab (REGN1500) is a monoclonal antibody against angiopoietin-like protein 3 (ANGPTL3) (29), a circulating protein that inhibits the hydrolysis of TGs by LPL in TG-rich lipoproteins. Loss-of-function mutations in the ANGPTL3 gene correlate with protection against CVD and treatment with evinacumab decreased plasma TG and LDL-C levels in human subjects (18, 30). Previous studies with atorvastatin (10 mg/kg/day) (16, 23, 31), alirocumab (10 mg/kg/week) (17), and evinacumab (25 mg/kg/week) (18) as mono-treatment in APOE*3-Leiden.CETP mice in an atherosclerosis prevention or progression mode showed reductions in plasma cholesterol (−53% to −71, −46, and −52%, respectively) and TGs (NS, −39%, and −84%), and strong reduction in development of atherosclerosis (−87, −88, and −39%, respectively). In this study, we tested alirocumab and/or evinacumab on top of atorvastatin as high-intensive lipid-lowering strategy to evaluate their effect on regression of pre-existent atherosclerosis in APOE*3-Leiden.CETP mice. In addition, we assessed the effects of the treatments on plaque composition and morphological changes of the plaque, and further looked into the process of macrophage reduction during regression.

## MATERIALS AND METHODS

### Animals and antibodies

Female APOE*3-Leiden.CETP transgenic mice on a C57BL/6 background (8–12 weeks of age) were obtained from the breeding facility of the Organization of Applied Scientific Research (TNO). The number of animals per group was calculated using a power of 0.80. Based on our experience from previous studies, we expected to have a variance of 23% in atherosclerosis, a minimal difference of 40%, and a two-sided test with 95% confidence interval, which resulted in 16 animals per group. Female mice were used as they are more susceptible to cholesterol-containing diets by having higher plasma cholesterol and TG levels relative to APOE*3-Leiden.CETP males, and therefore develop more pronounced atherosclerotic lesions (32). The mice entered the study in a staggered way of 5 weeks apart with two equal batches of eight mice each per group to limit the difference in animal age.

Alirocumab and evinacumab are fully human monoclonal antibodies derived using Regeneron’s Velocimmune® technology platform (33). Alirocumab recognizes mouse PCSK9 and evinacumab recognizes mouse ANGPTL3, with affinity comparable to the respective human proteins; both antibodies were previously evaluated in mouse models and dose selection was performed based on previous studies (17, 18, 29). The presence of antibodies in the circulation throughout the study was evaluated by human fragment crystallizable ELISA described in (18). Multiple administrations of human antibodies to mice often lead to development of mouse-anti-human antibodies and results in fast clearance of the human antibody from the circulation. Based on our previous knowledge, alirocumab does not cause auto-antibody development in mice, while around 25–40% of mice develop such a response after evinacumab administration (18). For this reason, additional mice per group were originally included in groups treated with evinacumab (32 for treatment with atorvastatin and evinacumab and 48 for atorvastatin, evinacumab, and alirocumab). All other groups had 16 mice per group at the beginning of the study. During the 38 week study with in total 144 mice, four mice were found dead in their cage and four mice were euthanized based on human end-point criteria (atorvastatin group: three mice; atorvastatin and alirocumab: two mice; atorvastatin and evinacumab: one mouse; atorvastatin, alirocumab, and evinacumab: two mice). In total, 48 mice developed auto-antibodies to evinacumab, as determined by ELISA described in (18) (atorvastatin and evinacumab: 18 mice; atorvastatin, alirocumab, and evinacumab: 30 mice) and were excluded from all analyses, allowing for 13–16 mice per group for atherosclerosis evaluation.

The study was performed at the research facility of TNO-Metabolic Health Research, The Netherlands, and animal experiments were approved by the Animal Experiment Committee of The Netherlands Organization of Applied Scientific Research TNO under registration number 3682.

### Diet and treatments

Mice were fed a Western-type diet (WTD) with 0.30% cholesterol and 15% saturated fat for 13 weeks to induce development of atherosclerosis. After 13 weeks, mice were matched into six groups based on age, body weight, plasma total cholesterol (TC) and TGs, and cholesterol exposure (millimoles per liter × weeks) measured at 12 weeks, and thereafter 16 mice were euthanized as the baseline control group (see supplemental Fig. S1 for study design). The other five groups continued to receive WTD alone or with treatment for 25 weeks: regression control, atorvastatin (5–13 mg/kg/day), atorvastatin and alirocumab (10 mg/kg/week), atorvastatin and evinacumab (25 mg/kg/week), or atorvastatin, alirocumab, and evinacumab. Atorvastatin was mixed with the diet in a dose of 5 mg/kg/day (weeks 13–14), 6 mg/kg/day (week 15), 13 mg/kg/day (weeks 16–24), and 7 mg/kg/day (weeks 25–38). However, the increase in atorvastatin dose led to increases in TG levels in all groups (starting from week 16), and, therefore, we decided to lower the atorvastatin dose at week 24. We decreased dietary cholesterol concentrations from 0.30% to 0.15% in week 24 for counterbalance. The lowering of dietary cholesterol resulted in TC levels of 11.5 mmol/l in control, which is a pro-atherogenic condition in APOE*3-Leiden.CETP mice (34). Alirocumab and evinacumab were administered by weekly subcutaneous injections.

Body weights, food intake per cage, and plasma parameters were measured throughout the study. To measure HDL-C, apoB-containing particles were precipitated from plasma with 20% polyethylene glycol in 200 mM glycine buffer (pH 10), and cholesterol was measured in the supernatant (26). Lipoprotein profiles from all individual mice were assessed by FPLC lipoprotein separation at end-point and the average profile is depicted (17). The development of atherosclerosis was analyzed at *t* = 13 weeks (baseline control group) and at *t* = 38 weeks (control and treatment groups) in the aortic arch and aortic root. Lesion severity was determined in the aortic root. Lesions were classified as mild lesions (types I–III according to the American Heart Association) (17, 35, 36) and complex lesions, which include type IV and V lesions (according to the American Heart Association) and the so-called “regression lesions”. Although the regression lesions were generally smaller than type IV and V lesions, they could not be defined as early fatty streak or mild lesions because they contained a low amount of macrophages and consisted mainly of collagen and smooth muscle cells containing α-actin (αSMCs).

Plaque composition, monocyte adherence, and macrophage proliferation were determined in the complex lesions of the aortic root. The stabilization/destabilization ratio is defined as the ratio of the stabilization factors, αSMC area in the fibrotic cap and collagen in the entire lesion, to the destabilization factors, macrophage and necrotic area, both in the entire lesion, and is calculated as described previously (37). The morphological changes described in this ratio are derived from human pathology where a vulnerable lesion is characterized by a thin collagen-poor fibrous cap, decreased SMCs, increased macrophage infiltration, and a large necrotic core (38). This type of lesion is referred to as a thin-cap fibroatheroma (39). Patients with unstable plaque have a higher incidence of new coronary events. The therapeutic target has, therefore, shifted from enlargement of the lumen toward stabilization of the plaque (40). Therefore, in addition to the lesion area, we also investigated the composition of the plaque by performing these histological analyses. Details of all antibodies used in the study are depicted in supplemental Table S1.

Detailed Materials and Methods information can be found in the supplemental material online.

### Statistical analysis

Significance of differences between the groups was calculated using one-way ANOVA followed by Dunnett’s two-sided post hoc test for comparisons against the control and baseline control group. The Bonferroni post hoc test was used to correct for multiple comparisons between the different treatment groups. For the atherosclerosis measurements, the nonparametric Kruskall-Wallis test was used to test for differences between groups, followed by a Mann-Whitney U test for comparisons against the baseline and control group and between the different treatment groups. Linear regression analyses were used to assess correlations between variables and the contribution of the cumulative decrease in plasma cholesterol and TG exposure to regression of atherosclerosis. IBM SPSS v24.0 was used for all analyses. *P* values ≤0.05 were considered statistically significant.

## RESULTS

### Double and triple treatment with alirocumab and evinacumab on top of atorvastatin gradually decreases TC and non-HDL-C

**Fig. 1.**
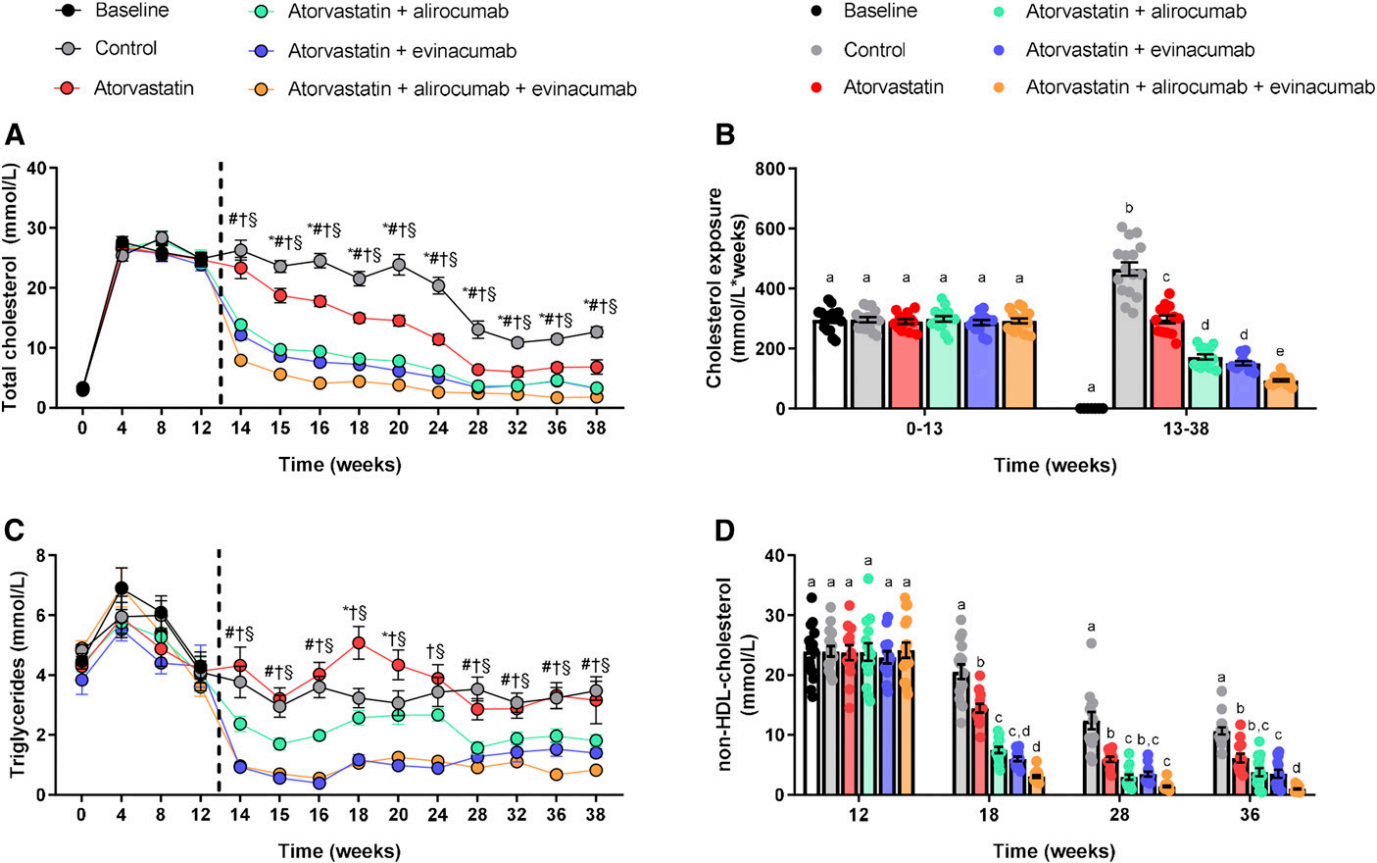
Double and triple treatment with alirocumab and evinacumab on top of atorvastatin gradually decrease TGs, TC, and non-HDL-C. APOE*3-Leiden.CETP mice were fed a WTD for 13 weeks to induce atherosclerosis and remained on the diet without or with treatment until end-point. Plasma TC (A), TC exposure (millimoles per liter × weeks) (B), plasma TG (C). Non-HDL (D) was calculated by subtracting HDL-C from TC. The dashed line represents start of treatment and euthanization of the baseline group. A, C: *P < 0.05 atorvastatin versus control, ^#^*P* < 0.05 atorvastatin + alirocumab versus control, †*P* < 0.05 atorvastatin + evinacumab versus control, §*P* < 0.05 atorvastatin + alirocumab + evinacumab versus control. B, D: Bars with different letters indicate statistical difference (*P* < 0.05). Data are presented as mean ± SEM (n = 13–16 per group).

Mice were fed WTD for 13 weeks, which led to increased plasma TC levels of about 25 mmol/l. At that point, the mice were matched into groups and treatment started. All treatments decreased plasma cholesterol (**Fig. 1A**) and cholesterol exposure (millimoles per liter × weeks) in comparison to control and atorvastatin (Fig. 1B), showing a gradual decline in the atorvastatin, double (alirocumab and atorvastatin, evinacumab and atorvastatin), and triple (alirocumab, evinacumab, and atorvastatin) treatment groups. Triple treatment lowered plasma TC levels to 1.8 mmol/l at the end-point and reduced cholesterol exposure by 80% (*P* < 0.001) relative to control, and by 68% (*P* < 0.001), 45% (*P* < 0.001), and 38% (*P* = 0.035) when compared with atorvastatin or double treatment with alirocumab or evinacumab, respectively. All treatments, except mono-treatment with atorvastatin, consistently decreased plasma TG levels (Fig. 1C), with the groups treated with evinacumab having the lowest TG exposure relative to control (−64%, *P* < 0.001 and −68%, *P* < 0.001), atorvastatin (−67%, *P* < 0.001 and −71%, *P* < 0.001), and double treatment with alirocumab (−48%, *P* = 0.006 and −55%, *P* < 0.001) (supplemental Fig. S2). Non-HDL-C levels were decreased by all treatments, with the largest reduction, down to 1.0 mmol/l, achieved by triple treatment at the end of the study (−91%, *P* < 0.001), which was significantly lower when compared with atorvastatin mono-treatment (−84%, *P* < 0.001) and double treatment with alirocumab (−74%, *P* = 0.010) and evinacumab (−72%, *P* = 0.033) (Fig. 1D). The reduction in TC was confined to the apoB-containing lipoproteins [VLDL (remnants) and LDL], showing decreases in both lipoprotein classes by the treatments. Atorvastatin, double treatment with alirocumab or evinacumab, and triple treatment reduced VLDL(remnant)-C by 45, 83, 87, and 95%, respectively, and LDL-C by 27 (NS), 53, 47, and 73%, respectively. There were no changes in HDL-C. (supplemental Fig. S3). Altogether, these data demonstrate that evinacumab on top of atorvastatin and alirocumab has an additional cholesterol-lowering effect resulting in non-HDL-C levels of 1 mmol/l.

### Triple treatment with alirocumab and evinacumab on top of atorvastatin regresses pre-existent lesions and reduces lipid content in the thoracic aorta

**Fig. 2.**
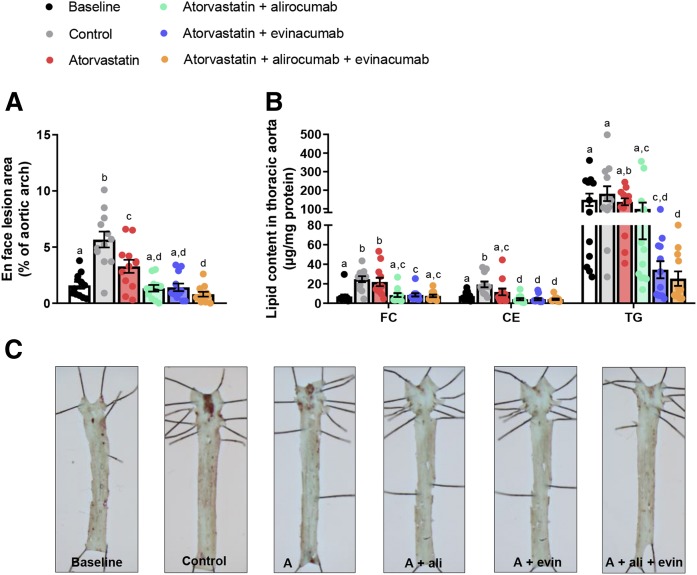
Triple treatment with alirocumab and evinacumab on top of atorvastatin regresses pre-existent lesions and reduces aortic lipid content in the thoracic aorta. En face analysis of atherosclerosis (A) and lipid content (B) in the thoracic aorta with representative images (C). Bars with different letters indicate statistical difference (*P* < 0.05). Data are presented as mean ± SEM (n = 12 per group). A, atorvastatin; ali, alirocumab; CE, cholesterol ester; evin, evinacumab; FC, free cholesterol.

We assessed the effect of intensive lipid lowering on the progression and regression of pre-existing atherosclerosis at different sites along the aorta, in the thoracic aorta and the aortic root. After 13 weeks of WTD (at treatment baseline), 1.6% of the thoracic aorta was covered with oil-red-O-positive lesions. WTD feeding for 25 more weeks led to further progression of atherosclerosis to 5.7% coverage in the control group. Atorvastatin reduced progression of atherosclerosis development and treatment with alirocumab or evinacumab on top of atorvastatin fully blocked progression (**Fig. 2A**, C). Double treatment with alirocumab or evinacumab decreased the amount of cholesterol ester, and double treatment with evinacumab decreased the TG content beyond baseline (Fig. 2B). Triple treatment not only blocked the progression (−86%, *P* < 0.001 vs. control) but also resulted in regression of the pre-existent lesions by 50% (*P* = 0.045) compared with baseline. Furthermore, triple treatment reduced cholesterol ester and TG content beyond the baseline level in the thoracic aorta (−45%, *P* = 0.033 and −83%, *P* = 0.001, respectively). The effect of triple and double treatments was stronger than atorvastatin mono-treatment on all parameters.

**Fig. 3.**
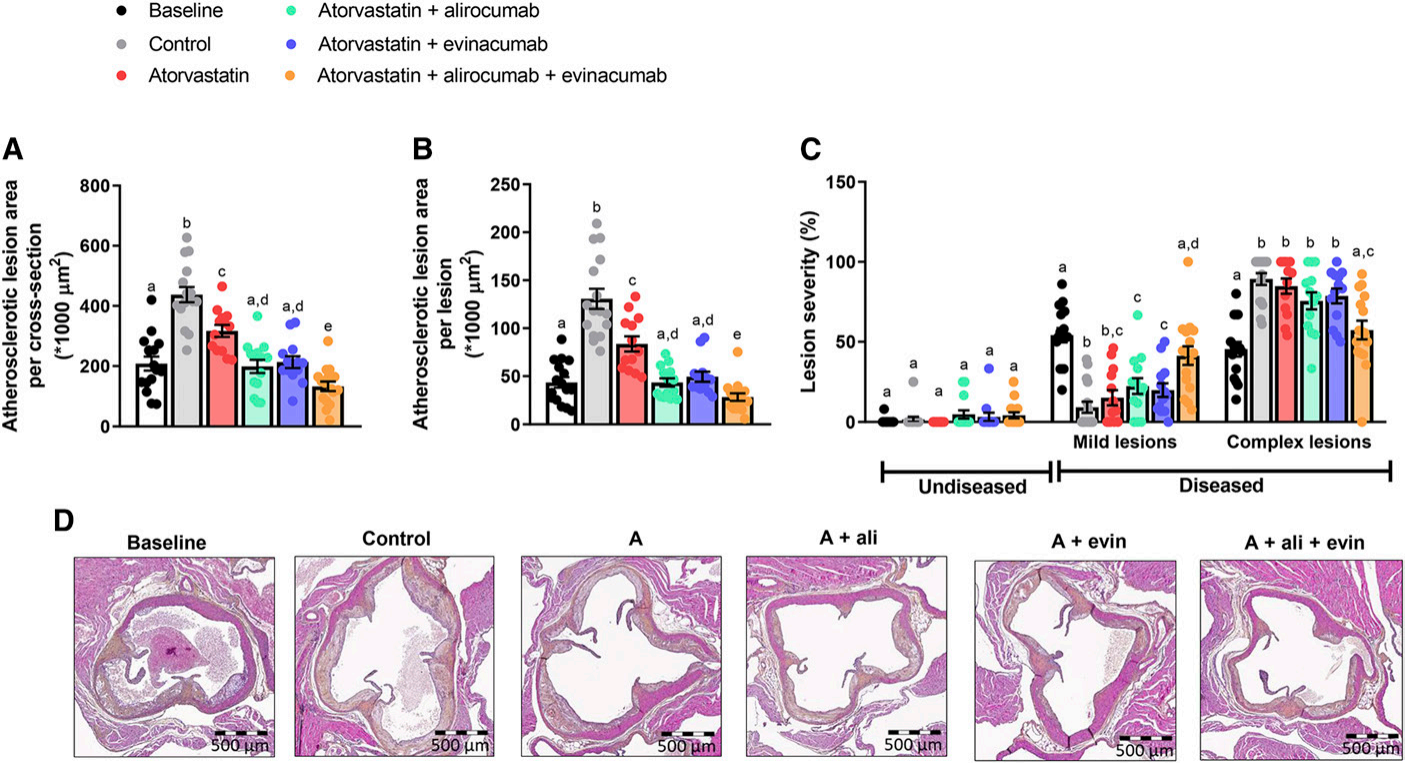
Double treatment with alirocumab or evinacumab on top of atorvastatin blocks the progression of atherosclerosis and triple treatment regresses pre-existent lesions in the aortic root. Lesion size in the aortic root after 13 weeks of WTD (baseline) and in control and treatment groups at end-point (week 38) (A). Number of lesions per cross-section was assessed and average size per lesion was calculated (B). Lesion severity as relative amount of mild and complex lesions together with lesion-free segments (C). Representative images (D). Bars with different letters indicate statistical difference (*P* < 0.05). Data are presented as mean ± SEM (n = 13–16 per group). A, atorvastatin; ali, alirocumab; evin, evinacumab.

In the aortic root, a 208 × 1,000 μm^2^ lesion area per cross-section was present at baseline, which further increased to a 438 × 1,000 μm^2^ lesion area in the control group. Atorvastatin modestly decreased lesion size (−28%, *P* = 0.001 vs. control), whereas double treatment with alirocumab or evinacumab on top of atorvastatin completely blocked the progression (−55%, *P* < 0.001; −51%, *P* < 0.001, respectively vs. control). Triple treatment further decreased lesion size (−70%, *P* < 0.001 vs. control) and regressed the atherosclerotic lesion size (−36%, *P* < 0.001 vs. baseline) (**Fig. 3A**). All treatments led to smaller lesions compared with control, and triple treatment lesions were smaller than the size of the initial lesions at baseline (Fig. 3B). The effect of triple and double treatments on lesion area was stronger than atorvastatin mono-treatment, and triple treatment lesions were smaller than double treatment lesions. The area that consisted of complex lesions was decreased by triple treatment compared with control (−36%, *P* < 0.001), with more mild lesions present (Fig. 3C). Additionally, triple treatment decreased lesion area and abated lesion severity as compared with mono-treatment and double treatment. Representative images of the aortic root area are shown in Fig. 3D. These data demonstrate that alirocumab and evinacumab on top of atorvastatin equally block the progression of atherosclerosis, but that regression of pre-existent advanced atherosclerotic plaques is only achieved by aggressive lipid lowering using triple combination treatment.

### The reduction in lesion size is correlated with the decrease in plasma cholesterol

**Fig. 4.**
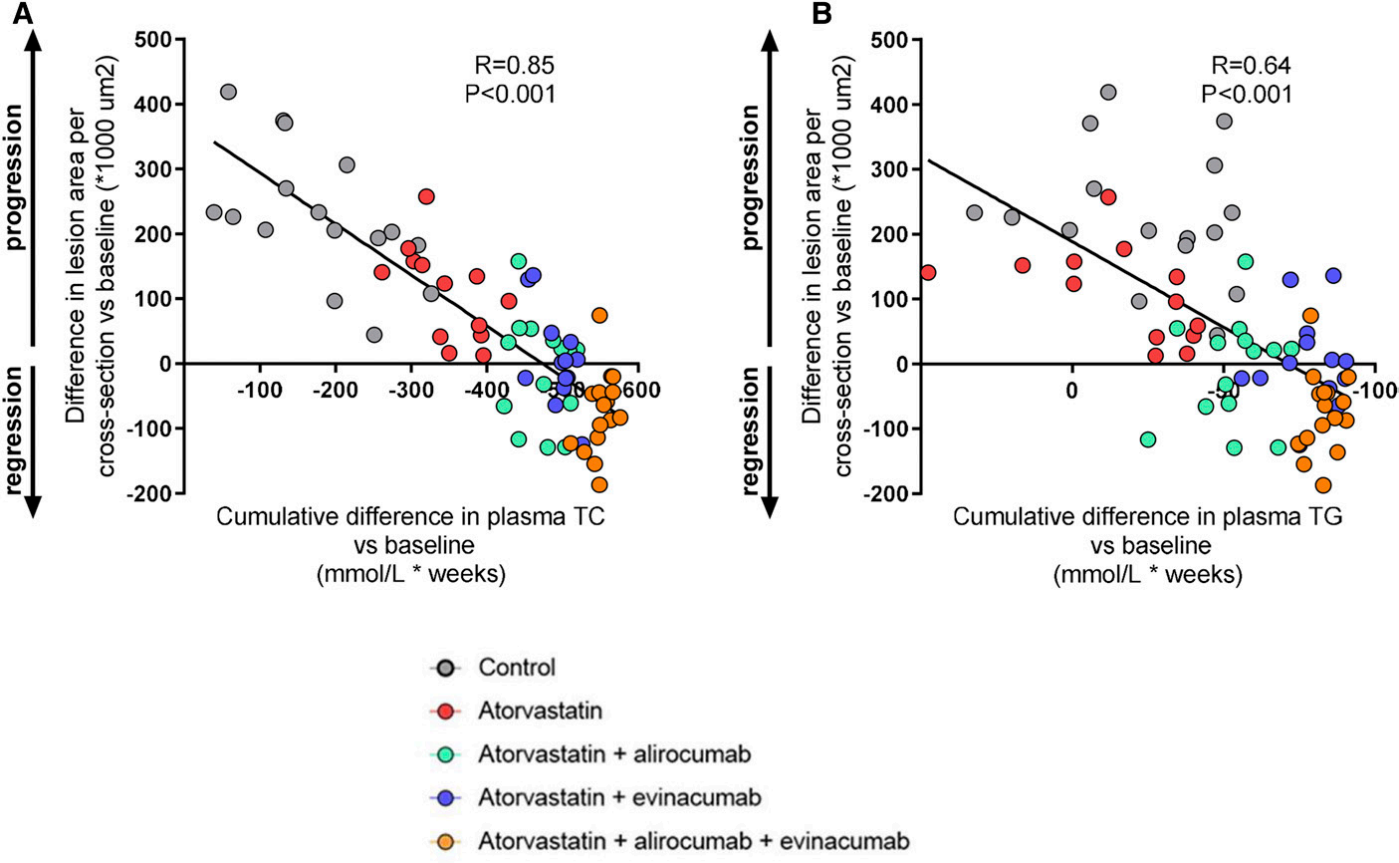
Correlation between the cumulative decrease in plasma cholesterol and TG exposure and atherosclerotic lesion area. Mean cholesterol or TGs at baseline was subtracted from cholesterol or TG levels of each individual mouse at each time point and the cumulative decrease in cholesterol (A) or TG (B) exposure during treatment was calculated as millimoles per liter × weeks. Data were plotted against the difference in lesion size at end-point and mean lesion size at baseline. Linear regression analysis was performed (n = 13–16 per group).

We evaluated whether the reduction in lesion size could be explained by the reduction in plasma TC and TGs during treatment. The mean TC or TG level at baseline was subtracted from the TC or TG levels of each individual mouse at each time point, and the cumulative decrease in cholesterol or TG exposure was calculated as millimoles per liter × weeks. These data were plotted against the lesion size at end-point minus the mean lesion size at baseline (**Fig. 4****)**. Univariate regression analysis showed a strong correlation (*R* = 0.85, *P* < 0.001) between the difference in lesion area and the cumulative TC decrease during treatment (Fig. 4A) and a marked correlation for the cumulative reduction in TGs (*R* = 0.64, *P* < 0.001) (Fig. 4B). However, assessment of the contribution of the cumulative decrease in plasma TC and TG exposure to regression of atherosclerosis revealed that the decrease in plasma TC versus baseline contributed significantly to the model (*P* < 0.001), whereas the decrease in plasma TGs versus baseline did not (*P* = 0.193). These data indicate an important role of therapeutic cholesterol lowering in lesion regression (*R*^2^ = 0.72, *P* < 0.001).

### Double and triple treatments improve plaque composition

**Fig. 5.**
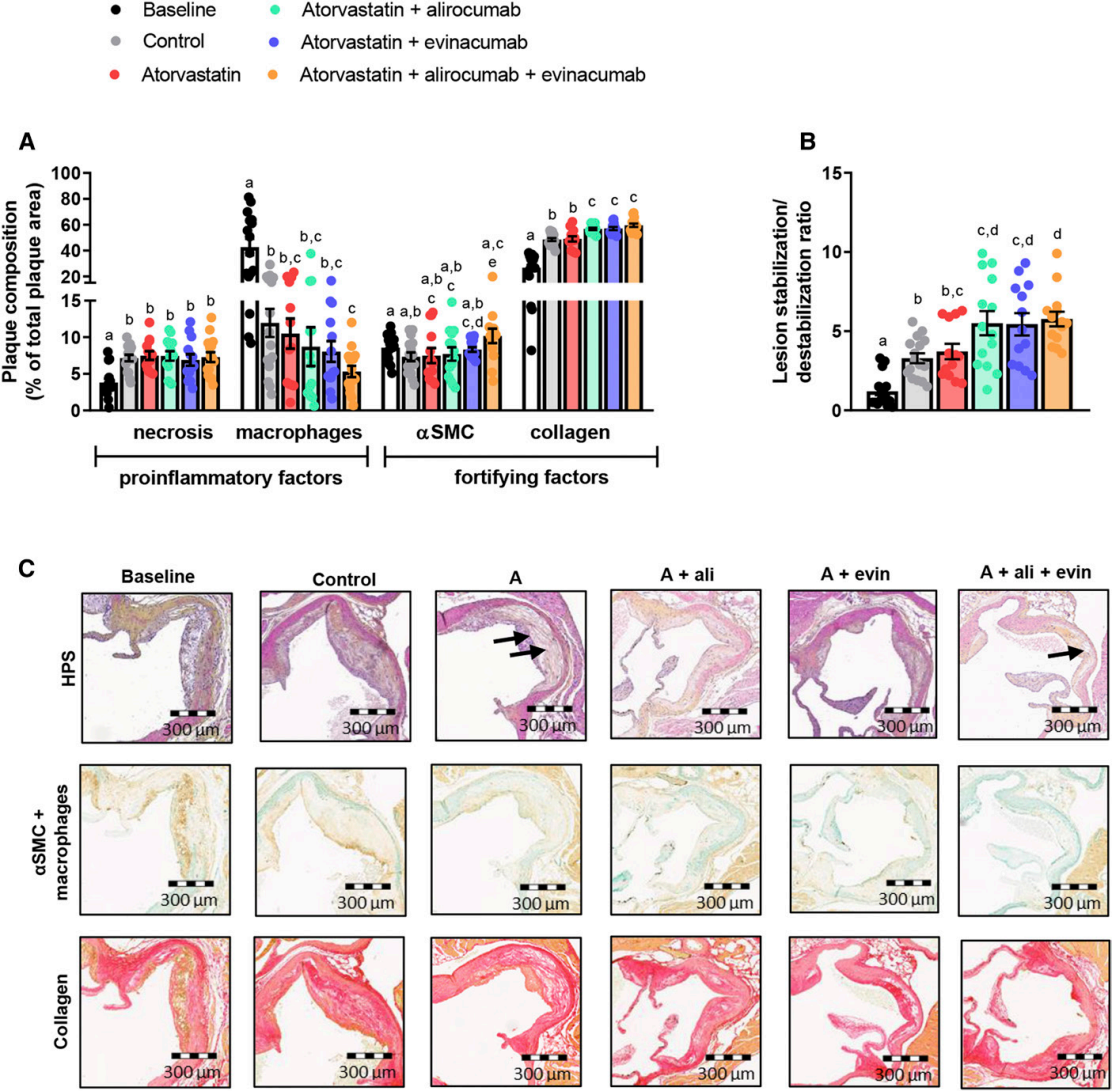
Double and triple treatments improve plaque morphology. Necrotic and macrophage content as pro-inflammatory factors and αSMCs and collagen as fortifying factors were determined in the complex lesions in the aortic root and expressed as a percentage of total plaque area (A). Lesion stabilization/destabilization ratio, as the ratio of collagen area and αSMC area in the cap (i.e., stabilization factors) to macrophage and necrotic area (i.e., destabilization factors), was calculated (B). Representative images of HPS staining, double-immunostaining with α-actin for SMCs (Vina green) and LAMP2 (M3/84) for macrophages (DAB, brown), and Sirius red staining for collagen. The arrows depict necrotic areas, including cholesterol clefts (C). Bars with different letters indicate statistical difference (*P* < 0.05). Data are presented as mean ± SEM (n = 13–16 per group). A, atorvastatin; ali, alirocumab; DAB, 3,3′-diaminobenzidine; evin, evinacumab; HPS, hematoxylin-phloxine-saffron; SMCs, smooth muscle cells.

To evaluate whether plaque composition was affected by the treatments, the necrotic core and the amount of macrophages, collagen, and αSMCs in the cap were quantified. Only triple treatment further decreased the macrophage content (−56%, *P* = 0.012) compared with control, in parallel with increased αSMC (+38%, *P* = 0.015) and collagen (+23%, *P* < 0.001) content (**Fig. 5A**). Double and triple treatment increased collagen content as compared with atorvastatin mono-treatment. The lesion stabilization/destabilization ratio improved with double (+66%, alirocumab and +64%, evinacumab, both *P* < 0.001) and triple (+74%, *P* < 0.001) treatment compared with control, and by triple treatment as compared with atorvastatin (+57%, *P* = 0.009) (Fig. 5B). There were no differences between double and triple treatment. Representative images are shown (Fig. 5C).

### Triple treatment reduces monocyte adherence and macrophage proliferation

**Fig. 6.**
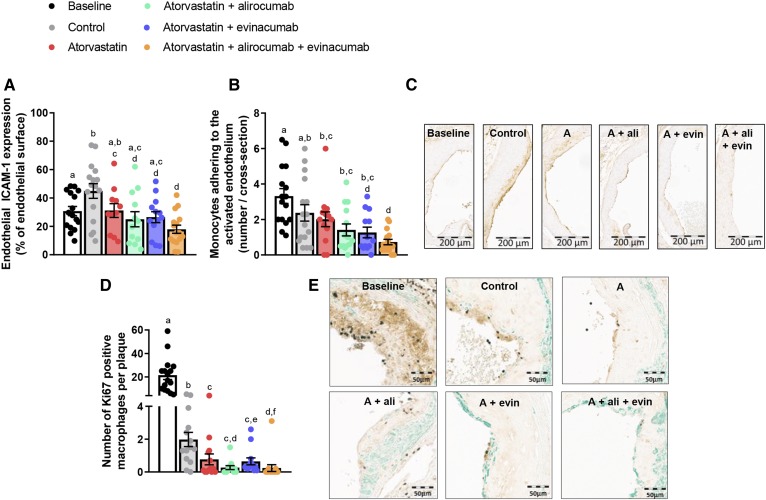
Monocyte adherence and the number of proliferative macrophages decrease in regression plaques. In each segment used for lesion quantification in the aortic root, endothelial ICAM-1 expression was determined as a percentage of the luminal surface (A). The number of monocytes adhering to the activated endothelium per cross-section after staining with AIA 31240 (B). Representative images of ICAM-1 expression (C). The number of Ki67-positive macrophages as marker for proliferation in type IV and type V plaques after triple immunostaining with Ki67 (DAB, black), LAMP2 (M3/84) for macrophages (DAB, brown), and α-actin for αSMC (green). The number of proliferative macrophages was counted per plaque (D). Representative images (E). Four mice were excluded from D (two mice in control, two mice in alirocumab + atorvastatin) due to extensive infiltration of the plaques by Ki67-positive inflammatory cells. Bars with different letters indicate statistical difference (*P* < 0.05). Data are presented as mean ± SEM (n = 11–16 per group). A, atorvastatin; ali, alirocumab; evin, evinacumab; DAB, 3,3′-diaminobenzidine; SMCs, smooth muscle cells.

Vascular inflammation is recognized to play an important role in both the initiation and progression of atherosclerosis, whereas proliferation of macrophages further increases the plaque burden. Therefore, we measured endothelial intercellular adhesion molecule 1 (ICAM-1) expression (41) and adherence of monocytes to the activated endothelium (42) as markers of vascular inflammation, and counted the number of currently proliferating macrophages after immunostaining for Ki67. All regimens except mono-treatment with atorvastatin decreased ICAM-1 expression when compared with control, but only triple treatment decreased ICAM-1 expression when compared with baseline (−37%, *P* = 0.010) (**Fig. 6A**, C). In addition, triple treatment decreased the number of monocytes adhering to the endothelium when compared with baseline (−78%, <0.001) and control (−69%, *P* = 0.003), whereas mono-treatment and double treatment only decreased monocyte adherence when compared with control (Fig. 6B). The number of proliferating macrophages per plaque (Fig. 6D) decreased over time by 91% (*P* < 0.001; control vs. baseline), which was further reduced by mono-treatment with atorvastatin (−60%, *P* = 0.019), double treatment with alirocumab or evinacumab on top of atorvastatin (−87%, *P* = 0.001 and −58%, *P* = 0.012 vs. control), and triple treatment (−88%, *P* < 0.001 vs. control). For all parameters, the effect of triple treatment was stronger than atorvastatin mono-treatment. Except for the number of proliferating macrophages in double treatment with evinacumab, there were no differences between double and triple treatment for these biomarkers. Representative images are shown (Fig. 6E).

## DISCUSSION

PCSK9 inhibition with alirocumab has been shown to strongly lower LDL-C and non-HDL-C alone and on top of statin, and reduce the risk of recurrent ischemic cardiovascular events in patients with acute coronary syndrome (28). The ANGPTL3 monoclonal antibody, evinacumab, was reported to reduce plasma TG and LDL-C levels in healthy subjects and patients with homozygous familial hypercholesterolemia (18, 30). Recent data suggest that not only LDL-C but also remnant cholesterol, and thus all non-HDL-C or apoB-containing lipoproteins, are important predictors of cardiovascular outcome (9–11). The present study was designed to investigate the effects of gradual and aggressive reduction of cholesterol in all apoB-containing lipoproteins (both remnant lipoproteins and LDL) by alirocumab and/or evinacumab on top of atorvastatin on regression of pre-existent atherosclerosis in hyperlipidemic mice. Our data revealed that alirocumab and evinacumab in combination with atorvastatin fully block further progression of atherosclerosis and triple treatment reduces lesion size beyond the treatment baseline level. In addition, double and triple treatments improve lesion morphology and composition in APOE*3-Leiden.CETP mice with pre-existent atherosclerosis. This is the first study in mice using the combination of clinical hypolipidemic drugs that shows true regression of atherosclerosis, not only macrophage content but also total lesion size, while mice were kept on the WTD during both progression and regression phases.

Therapeutic interventions in mice have been hampered due to the lack of responsiveness to current lipid-lowering therapies in murine models of regression. Commonly used models are the aortic transplant model, the Reversa mouse (*Ldlr*^−/−^*^Apob^*^100/100^*^Mttp^*^fl/fl^Mx1*Cre*^+/+^), and *Apo*e^−/−^ and *Ldlr*^−/−^ mice [reviewed in (14, 43)]. In these models, progression of atherosclerosis is induced by a WTD, and regression is accomplished by a switch to chow, eventually together with genetic alterations or treatment strategies. Regression of atherosclerosis was generally defined by a decrease of macrophages or lipid content (14, 43), though some studies reported a reduced total lesion size using experimental interventions, which was independent of plasma TC levels [reviewed in (14, 43)]. While these models are of great value to elucidate the molecular characteristics of the regressive plaque, they are less suitable for the evaluation of lipid-lowering interventions and their effect on atherosclerosis regression, as they poorly respond to registered lipid-lowering drugs (19, 27). In the present study, we used the APOE*3-Leiden.CETP mouse, which possesses a delayed but intact apoE-LDLR-mediated clearance pathway and expresses cholesteryl ester transfer protein (CETP) (15, 27). These mice respond well to lipid-lowering drugs used in the clinic with respect to their effects on plasma cholesterol and TGs, including statins, alirocumab, and evinacumab (16–21). Thus, treatments of APOE*3-Leiden.CETP mice on WTD with atorvastatin or alirocumab on top of the statin in our study decreased TC levels (−31% to −51% vs. control and −32% to −52% vs. atorvastatin, respectively) by a reduction of non-HDL-C, similarly as in humans (28, 44). Evinacumab has an additive effect on treatment with atorvastatin and alirocumab by further reducing TC (−62% to −75% vs. atorvastatin; −34% to −63% vs. atorvastatin + alirocumab) and, in addition, TG levels (−62% to −80% vs. atorvastatin; −42% to −65% vs. atorvastatin + alirocumab).

We have previously shown that the lipid-modifying effects of PCSK9 and ANGPTL3 inhibition have an atheroprotective effect in a preventive design (17, 18). However, to date, the effect of pharmacological inhibition of PCSK9 and ANGPTL3 on regression of atherosclerosis has not been investigated. Here, we show for the first time that double treatment with alirocumab or evinacumab on top of atorvastatin completely blocks progression of pre-existent atherosclerosis and that triple treatment regresses atherosclerosis in the aortic arch and the aortic root. The treatment effects on lesion area were mainly predicted by the gradual and aggressive reduction in plasma TC levels as illustrated by the strong association between the decreased cholesterol exposure and lesion size during treatment (*R* = 0.85), indicating an important role of therapeutic cholesterol lowering in lesion regression (*R*^2^ = 0.72). All triple-treated mice except one showed a lesion size below that at baseline, indicating regression. Based on our data, reduction of non-HDL-C levels to about 1 mmol/l (38.7 mg/dl) was required to observe the regression.

Vulnerable plaques with high macrophage content, a large necrotic core, and a thin collagen-poor fibrous cap are more prone to rupture (38–40, 45). Thus, lesion composition, not just lesion size, is another important characteristic of the plaque. In the present study, the decline in plasma cholesterol reduced the lipid content of the aorta and resulted in smaller and less inflamed lesions. Double and triple treatment decreased endothelial expression of ICAM-1 and consequently reduced monocyte adhesion to the activated vascular endothelium, well-recognized processes in the initiation of atherosclerosis. In hypercholesterolemia, modified lipoproteins induce endothelium activation, thereby mediating the arrest and transmigration of circulating monocytes into the subendothelial space where they differentiate into macrophages (46). All treatments and control in the present study reduced the macrophage content as compared with baseline, only triple treatment reduced macrophage content as compared with control, and double and triple treatment increased the amount of collagen in the lesions, resulting in an improved plaque morphology. The large reduction in macrophage content in the present and other studies is a key feature of regression and depends on the balance between recruitment of monocytes and their differentiation into macrophages, proliferation of macrophages, and apoptosis and migratory egress from the plaques. However, whereas impaired monocyte transmigration during the initiation of atherosclerosis diminishes plaque volume (47), monocyte depletion per se does not affect further progression of plaque burden (48). Local proliferation of aortic macrophages has been reported to be a key event in the progression of atherosclerosis and to substantially contribute to lesional macrophage accumulation (48). Here, we provide evidence that cholesterol lowering-induced regression decreases the number of Ki67-positive macrophages, a marker of currently proliferating macrophages. This finding indicates that diminished proliferation of macrophages contributes to the reduction in macrophage content during regression of atherosclerosis.

In conclusion, we show that high-intensive lipid-lowering triple treatment with atorvastatin, alirocumab, and evinacumab regresses atherosclerosis, abates lesion severity, and reduces the proliferation of macrophages in the plaques. These data show that further reduction of plasma cholesterol together with TG lowering to target all apoB-containing lipoproteins may be an effective approach to further reduce existing atherosclerosis in dyslipidemic patients at cardiovascular risk resulting in a further decrease of clinical events and an increase of symptom-free years.

## Supplementary Material

Supplemental Data
