# Supplementary material for: Alirocumab, evinacumab, and atorvastatin triple therapy regresses plaque lesions and improves lesion composition in mice
Source: J Lipid Res. 2019 Dec 16;61(3):365–75. doi: 10.1194/jlr.RA119000419 (PMC7053846; doi:10.1194/jlr.RA119000419)
Supplement: Supplemental Data [file supp_61_3_365__index.html]

Alirocumab, evinacumab, and atorvastatin triple therapy regresses plaque lesions and improves lesion composition in mice — Cholesterol-lowering triple treatment regresses lesion size — Alirocumab, evinacumab, and atorvastatin triple therapy regresses plaque lesions and improves lesion composition in mice — Supplemental Data 

# Alirocumab, evinacumab, and atorvastatin triple therapy regresses plaque lesions and improves lesion composition in mice

## Supplemental Data

- Alirocumab, evinacumab, and atorvastatin triple therapy regresses plaque lesions and improves lesion composition in mice - Supplemental data Pouwer et al.
