# Supplementary material for: Alirocumab, evinacumab, and atorvastatin triple therapy regresses plaque lesions and improves lesion composition in mice
Source: J Lipid Res. 2019 Dec 16;61(3):365–75. doi: 10.1194/jlr.RA119000419 (PMC7053846; doi:10.1194/jlr.RA119000419)
Supplement: Supplemental Data [file supp_RA119000419_156034_2_supp_443444_q2lpl2.pdf]

Supplementary material

Belonging to the manuscript entitled "Alirocumab, evinacumab, and atorvastatin triple therapy regresses plaque lesions and improves lesion composition in mice" by Marianne G. Pouwer, Elsbet J. Pieterman, Nicole Worms, Nanda Keijzer, J. Wouter Jukema, Jesper Gromada, Viktoria Gusarova, Hans M. G. Princen.

Materials and methods

Plasma lipids

Plasma total cholesterol (TC) and triglycerides (TG) were determined at week 0, 4, 8, 12, 14, 15, 16, 20, 24, 28, 32, 36 and 38 using enzymatic colorimetric methods (Roche Diagnostics GmbH, Germany) according to the manufacturer's protocols and total cholesterol exposure was calculated as mmol/L*weeks. HDL-C was measured at week 12, 18, 28 and 36 after precipitation of apoB-containing particles as described previously (1), and non-HDL was calculated by subtracting HDL-C from total cholesterol. Briefly, to measure HDL-C, apoB-containing particles were precipitated from diluted plasma (15 µL previously frozen plasma + 15 µl PBS) by adding 5 µL of 20% polyethylene glycol (PEG) in 200 mM glycine buffer (pH10). This mixture was incubated for 5 min at 25°C and centrifuged at 6000 rpm for 5 min at 25°C. Thirty µL of supernatant was mixed with 20 µl of 20% PEG in 200 mM glycine buffer and incubated for 5 min at 25°C and centrifuged at 6000 rpm for 20 min at 25°C. TC was measured in the supernatant to determine plasma HDL-C levels (1).

En face determination of atherosclerosis in the thoracic aorta

To determine the total plaque load in the aortic arch, perfusion-fixed aortas (from the aortic origin to the diaphragm) were cleaned of extravascular fat, opened longitudinally, pinned en face, and stained for lipids with oil-red O (Sigma-Aldrich Chemie BV) as described previously (2). Photographs of the aorta's were taken by an Olympus SZX10 microscope with an Olympus DP74 camera. Data were normalized for the analyzed surface area and expressed as percentage of the stained area.

Determination of lipid content in the thoracic aorta

The thoracic aortas were cleaned of extravascular fat, homogenized in phosphate-buffered saline, and the protein content was measured using a Lowry protein assay. Lipids were extracted as described previously (3), separated by high-performance thin-layer chromatography on silica gel plates, stained

and analyzed with ChemiDoc Touch Imaging System (Bio-Rad). TG, cholesterol ester (CE) and free cholesterol (FC) content were quantified using Image-lab version 5.2.1 software (Bio-Rad) and expressed per mg protein.

Histological assessment of atherosclerosis in the aortic root

Atherosclerotic lesion area and severity were assessed in the aortic root area, as reported previously (4). Briefly, the aortic root was identified by the appearance of aortic valve leaflets, and serial cross-sections of the entire aortic root area (5 μm thick with intervals of 50 μm) were mounted on slides and stained with haematoxylin-phloxine-saffron (HPS). For each mouse, the lesion area was measured in 4 subsequent sections. Each section consisted of 3 segments (separated by the valves). The total lesion area and number of lesions were calculated per cross-section. Lesion severity was calculated as relative amount of mild and complex lesions in which the lesion-free segments are included. The lesions were classified as mild lesions (type I-III according to the American Heart Association (AHA)) and complex lesions, which include type IV-V lesions (according to the AHA (5)) and the so-called 'regression lesions'. Although the 'regression lesions' were generally smaller than type IV and V lesions, they could not be defined as mild lesions/fatty streak since they did not consist of macrophages, but mainly of collagen and αSMCs. Slides were scanned by an Aperio AT2 slide scanner (Leica Biosystems) and atherosclerotic area was measured in Image Scope (version 12-12-2015).

Histological assessment of plaque composition

Lesion composition in complex lesions was assessed after double immunostaining with anti-α smooth muscle actin (1:400; PROGEN Biotechnik GmbH, Germany) for smooth muscle cells (SMC), and anti-mouse LAMP2 (M3/84) (1:500; BD Pharmingen, the Netherlands) for macrophages. Anti-α smooth muscle actin was labeled with Vina green (Biocare Medical, Pacheco, USA), and LAMP2 with DAB (Vector laboratories, Burlingame, USA). After slides were scanned and analyzed, cover slips were detached overnight in xylene and Sirius Red staining for collagen was performed. Color intensity of

Sirius red staining was determined in ImageJ and the used threshold was confirmed by evaluation of the sections under polarized light. The necrotic area and cholesterol clefts were measured in the Sirius Red-stained slides. Lesion stabilization/destabilization ratio , as the ratio of collagen and αSMC area in the cap (i.e. stabilization factors) to macrophage and necrotic area (i.e. destabilization factors) was calculated as described previously (4). In each segment used for lesion quantification, ICAM-1 expression and the number of monocytes adhering to the activated endothelium were counted after immunostaining with mouse monoclonal ICAM-1 antibody (1:400; Santa Cruz Biotechnology, Dallas, USA) and AIA 31240 antibody (1:500; Accurate Chemical and Scientific, New York, USA), respectively (6). The number of proliferating macrophages in the plaques was counted after triple staining with Ki67 (1:1600, Abcam, Cambridge, UK) for cellular proliferation labeled with DAB (black) (Vector laboratories, Burlingame, USA), anti-mouse LAMP2 (M3/84) (1:500; BD Pharmingen, the Netherlands) for macrophages labeled with DAB (brown) (Vector Laboratories, Burlingame, USA) and anti-α smooth muscle actin (1:400; PROGEN Biotechnik GmbH, Germany) labeled with Vina green (Biocare Medical, Pacheco, USA). Slides were scanned by an Aperio AT2 slide scanner (Leica Biosystems). Monocyte adherence, ICAM-1 expression and the number of Ki67 positive macrophages were assessed in Image Scope (version 12-12-2015), and plaque composition was measured in Fiji (version 30-5-2017). Details of the antibodies used in the study are depicted in **Supplemental Table I**.

Supplementary table

Supplemental Table S1. Details of the antibodies used in the study.

Marker	Antibody	Dilution	Company	Product number
αSMC	Alpha-smooth muscle actin mouse monoclonal, IgG	1:400	Progen	61001
Macrophages	Rat monoclonal to LAMP2 antibody (M3/84), IgG	1:500	ThermoFisher Scientific	MA5-17861
Monocytes	Rabbit anti-mouse AIA 31240	1:500	Accurate Chemical & Scientific Corp.	J1857
ICAM-1	Mouse monoclonal to ICAM-1, IgG	1:400	Santa Cruz Biotechnology	Sc-8439
Ki-67	Rabbit polyclonal to Ki-67	1:1600	Abcam	ab15580

αSMC, alpha-smooth muscle cell actin; LAMP2, lysosome-associated membrane protein 2; ICAM-1, intercellular adhesion molecule 1

Supplementary figures

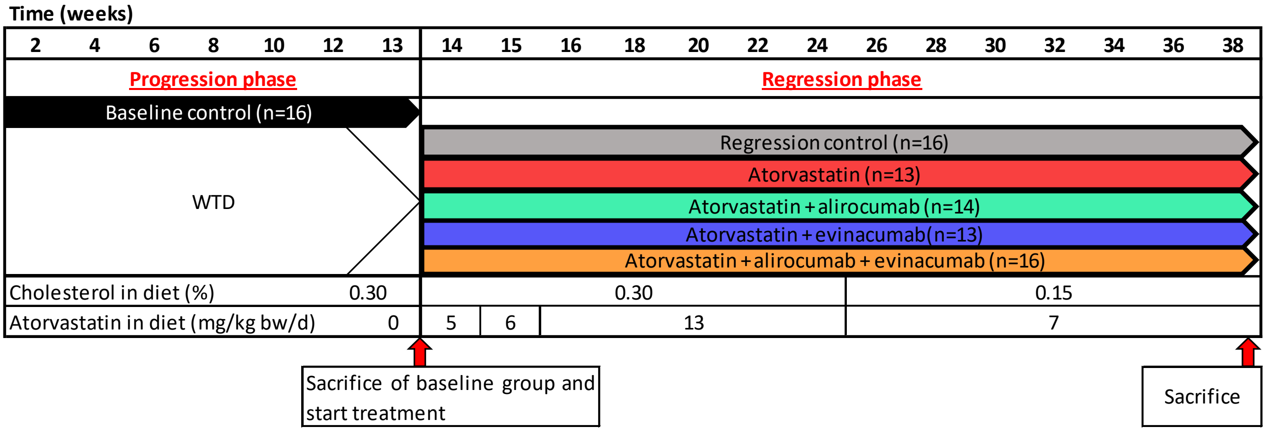

Supplemental Figure S1. Study design. Female APOE*3-Leiden.CETP mice were fed a Western type (WTD) diet for 13 weeks. Next, mice were matched in 6 groups based on age, body weight, plasma total cholesterol, triglycerides and cholesterol exposure (mmol/L*weeks). The baseline control group was sacrificed at t=13 weeks and the other 5 groups continued to receive a WTD alone (regression control) or with treatment as indicated for 25 weeks until week 38. The number of mice used for the analyses are depicted, this number excludes the mice that died during the study and mice that were excluded because of development of auto-antibodies to the human monoclonal antibody evinacumab (see Materials and Methods section).

WTD, Western type diet.

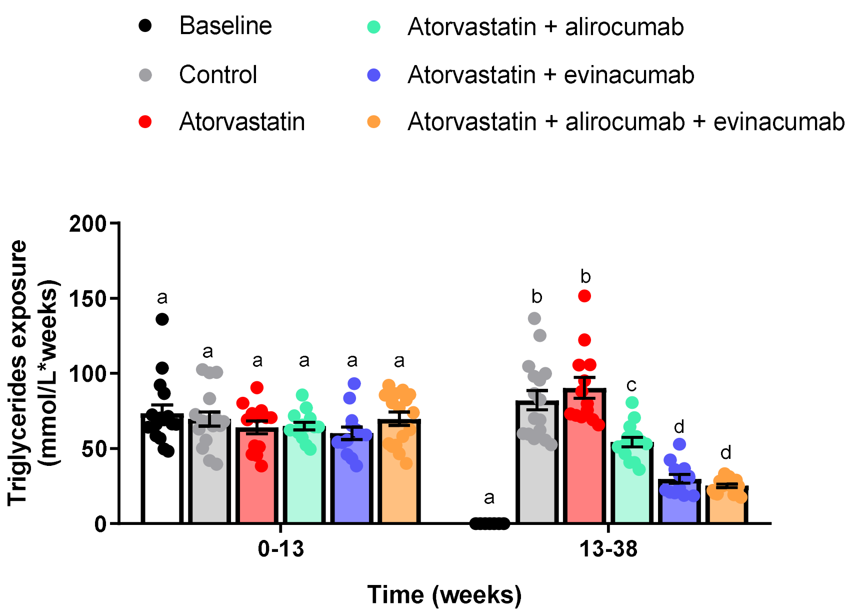

Supplemental Figure S2. Total triglycerides exposure (mmol/L*weeks).

Bars with different letters indicate statistical difference (P<0.05). Data are presented as means ± SEM (n=13-16 per group).

The groups treated with evinacumab reduced TG exposure relative to control by 64% (P<0.001) and 68% (P<0.001), when compared to atorvastatin by 67% (P<0.001) and 71% (P<0.001) and as compared to double treatment with alirocumab by 48% (P=0.006) and 55% (P<0.001).

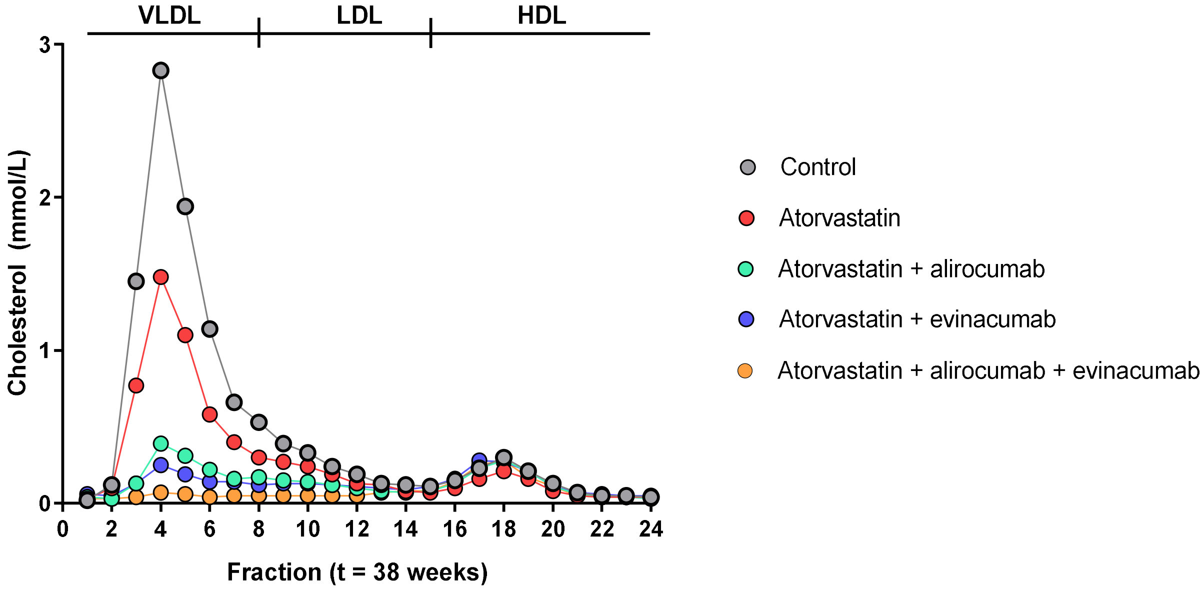

Group	VLDL(remnants)-C mmol/L	LDL-C mmol/L
Control	8.7 ± 2.8[a]	1.5 ± 0.5[a]
Atorvastatin	4.8 ± 3.8[b]	1.1 ± 0.9[a,b]
Atorvastatin + alirocumab	1.5 ± 0.9[c]	0.7 ± 0.5[b,c]
Atorvastatin + evinacumab	1.1 ± 0.7[c]	0.8 ± 0.5[b,c]
Atorvastatin + alirocumab + evinacumab	0.4 ± 0.2[c]	0.4 ± 0.3[c]

Supplemental Figure S3. Lipoprotein profiles and cholesterol content in individual lipoproteins at end-point. APOE*3-Leiden.CETP mice were fed a WTD for 13 weeks to induce atherosclerosis and remained on the diet without or with treatment until end-point (t = 38 weeks). Lipoprotein profiles from all individual mice were assessed by FPLC lipoprotein separation at end-point (n=13-16 per group) and the average profiles are depicted in the figure (7). For sake of clarity no error bars are shown in the **Figure**. Cholesterol (C) content contained in the VLDL(remnants) (fractions 1-8) and LDL fractions (fractions 9-15) were calculated and are given in the **Table**. Bars with different letters indicate statistical difference (P<0.05). Data are presented as means ± SEM (n=13-16 per group).

VLDL, very-low density lipoprotein; LDL, low-density lipoprotein; HDL, high-density lipoprotein; WTD, Western type diet; FPLC, fast protein liquid chromatography**.** N.S., not significant.

Atorvastatin, double treatment with alirocumab or evinacumab and triple treatment reduced VLDL(remnant)-C by 45% (P<0.001), 83% (P<0.001), 87% (P<0.001) and 95% (P<0.001), respectively, and LDL-C by 27% (N.S.), 53% (P=0.002), 47% (P=0.005) and 73% (P=0.001), respectively. As compared to atorvastatin dual and triple treatment decreased VLDL(remnants)-C and triple treatment decreased LDL-C. There were no changes in HDL-C (fractions 16-24).

References

1. Kühnast, S., S.J.L. van der Tuin, J.W.A. van der Hoorn, J.B. van Klinken, B. Simic, E. Pieterman, L.M. Havekes, U. Landmesser, T.F. Lüscher, K. Willems van Dijk, P.C.N. Rensen, J.W. Jukema, and H.M.G. Princen. 2015. Anacetrapib reduces progression of atherosclerosis, mainly by reducing non-HDLcholesterol, improves lesion stability and adds to the beneficial effects of atorvastatin. Eur. Heart J. 36: 39-48.

2. Groot, P.H., B.J. van Vlijmen, G.M. Benson, M.H. Hofker, R. Schiffelers, M. Vidgeon-Hart, and L.M. Havekes. 1996. Quantitative assessment of aortic atherosclerosis in APOE*3-Leiden transgenic mice and its relationship to serum cholesterol exposure. Arterioscler. Thromb. Vasc. Biol. 16: 926–933.

3. Post, S.M., J.P. Zoeteweij, M.H. Bos, E.C. de Wit, R. Havinga, F. Kuipers, and H.M. Princen. 1999. Acyl-coenzyme A:cholesterol acyltransferase inhibitor, avasimibe, stimulates bile acid synthesis and cholesterol 7alpha-hydroxylase in cultured rat hepatocytes and in vivo in the rat. Hepatology 30: 491–500.

4. Kühnast, S., J.W.A. van der Hoorn, A.M. van den Hoek, L.M. Havekes, G.W. Liau, J.W. Jukema, and H.M.G. Princen. 2012. Aliskiren inhibits atherosclerosis development and improves plaque stability in APOE*3Leiden.CETP transgenic mice with or without treatment with atorvastatin. J. Hypertens. 30: 107-116.

5. Stary, H.C., A.B. Chandler, R.E. Dinsmore, V. Fuster, S. Glagov, W.J. Insull, M.E. Rosenfeld, C.J. Schwartz, W.D. Wagner, and R.W. Wissler. 1995. A definition of advanced types of atherosclerotic lesions and a histological classification of atherosclerosis. A report from the Committee on Vascular Lesions of the Council on Arteriosclerosis, American Heart Association. Circulation. 92: 1355–1374.

6. Landlinger, C., M.G. Pouwer, C. Juno, J.W.A. van der Hoorn, E.J. Pieterman, J.W. Jukema, G. Staffler, H.M.G. Princen, and G. Galabova. 2017. The AT04A vaccine against proprotein

convertase subtilisin/kexin type 9 reduces total cholesterol, vascular inflammation, and atherosclerosis in APOE*3Leiden.CETP mice. Eur. Heart J. 38: 2499–2507.

7. Kühnast, S., J.W.A. van der Hoorn, E.J. Pieterman, A.M. van den Hoek, W.J. Sasiela, V. Gusarova, A. Peyman, H.L. Schäfer, U. Schwahn, J.W. Jukema, and H.M. Princen. 2014. Alirocumab inhibits atherosclerosis, improves the plaque morphology, and enhances the effects of a statin. J. Lipid Res. 55: 2103–2112.
